# Expressions of psychological distress in Sierra Leone: implications for community-based prevention and response

**DOI:** 10.1017/gmh.2020.12

**Published:** 2020-07-29

**Authors:** Rebecca Horn, Simeon S. Sesay, Mamadu Jalloh, Amjata Bayoh, Joan B. Lavally, Alastair Ager

**Affiliations:** 1 NIHR Global Health Research Unit on Health in Situations of Fragility, Institute for Global Health & Development, Queen Margaret University, Musselburgh, Edinburgh, Scotland; 2 Independent researcher, Freetown, Sierra Leone

**Keywords:** Cultural idioms, mental health, prevention, psychological distress, Sierra Leone, well-being

## Abstract

**Background:**

Over recent decades there has been considerable mental health research in Sierra Leone but little on local conceptualisations of mental health conditions. Understanding these is crucial both for identifying the experienced needs of the population and utilising relevant community-based resources to address them. This study took a grounded approach to identify the ways in which adults in Sierra Leone express psychological distress.

**Methods:**

Rapid ethnographic methods deployed included 75 case study interviews with community members, 12 key informant (KI) pile sorts and 55 KI interviews. Thematic analysis of data was supported by frequency analysis and multi-dimensional scaling.

**Results:**

Thirty signs of distress were identified. The only consistent ‘syndrome’ identified with respect to these was a general concept of *crase*, which referred to psychosis-related presentation but also a wide range of other signs of distress. We did not find consensus on locally defined concepts for mild-moderate forms of mental disorder: people use multiple overlapping signs and terms indicating psychological distress.

**Conclusions:**

Analysis supports calls to view mental health problems as a ‘continuum of distress’ rather than as discrete categories. This framing is coherent with opportunities for prevention and response in Sierra Leone which do not focus primarily on formal healthcare service providers but rather involve a range of community-based actors. It also enables attention to be paid to the identification of milder signs of distress with a view to early response and prevention of more severe mental health problems.

## Introduction

Understanding cultural idioms of distress (common modes of expressing distress within a specific cultural or social context) and local explanatory models (ways that people explain and make sense of their symptoms or illness) is important for the development of culturally-relevant and acceptable mental health services and supports (Nichter, 1981; Kirmayer and Bhugra, 2009).

Studies have found important variance in the ways that people express distress in different contexts (e.g. Keys *et al*., 2012; Haroz *et al*., 2017; Widiana *et al*., 2018; Housen *et al*., 2019). Yet, the assumption noted by Kleinman (1977), that because phenomena can be identified in different cultural settings they have the same meaning across these settings, continues to be evident in many studies. For example, in their systematic review of the emic literature on post-traumatic stress disorder (PTSD) outside North America and Europe, Rasmussen *et al*. (2014) found that most research was based on instruments measuring pre-defined symptoms from international classification systems such as the DSM or ICD, without exploring whether these symptoms were relevant to local expressions of distress following traumatic events or examining whether there might be symptoms not included in these measures that might also be important.

Emic approaches identify specific ways in which psychological distress is experienced and expressed in various cultural contexts (Miller *et al*., 2006), and global mental health practitioners have demonstrated that it is possible to effectively integrate such understandings of distress with western psychiatric approaches (Patel *et al*., 2018). There are also significant advantages in doing so. Understanding local illness models and idioms of distress facilitates both effective measurement of psychosocial wellbeing and mental health (Bass *et al*., 2007) and the design of support services that meet the needs of the population (including formal and informal medical systems, and religious and community resources, e.g. Bolton *et al*., 2013; Hassan *et al*., 2015; Cavallera *et al*., 2016; WHO, 2017,).

## Understanding mental health in Sierra Leone

The West African country of Sierra Leone experienced a brutal civil war between 1991 and 2002, during which an estimated 70,000 people were killed and more than 2 million (more than one-third of the population) were displaced (Kaldor and Vincent, 2006). Following the war, efforts were made to rebuild systems and infrastructure within Sierra Leone, but these efforts were disrupted by the outbreak in 2014 of Ebola virus disease (EVD) which continued for almost 2 years, and had a devastating effect on an already fragile population. One year after the end of the EVD outbreak the capital city, Freetown, was hit by a deadly mudslide and catastrophic flooding.

The majority of published research on mental health in Sierra Leone relates to the impact of emergency situations, particularly the prolonged civil conflict and the EVD outbreak (Shackman and Price, 2013; Bah *et al*., 2018), with few studies focusing on mental health in the general population outside of an emergency context.

### Mental health research related to the civil war

There are conflicting findings about the impact of the war in Sierra Leone on mental health (Shackman and Price, 2013). Some researchers reported increases in mental health conditions amongst the population affected by the civil war, including anxiety, drug abuse, schizophrenia, depression and PTSD (de Jong *et al*., 2000; Fox and Tang, 2000; Asare and Jones, 2005), and studies of children forcibly recruited into armed groups in Sierra Leone have found high rates of depression, anxiety and PTSD (Betancourt *et al*., 2010*a*, 2010*b*). World Health Organization's (WHO) pilot epidemiological survey conducted 1 year after the end of hostilities (Jensen and Nahim, 2002; Alemu *et al*., 2012) found the prevalence rates for severe mental illness to be 2% for psychosis, 4% for severe depression and 4% for severe substance abuse. Research related to the mental health impacts of the civil war has been highly focused on identifying PTSD, as is often the case in research on conflict-affected populations.

### Mental health research related to the Ebola outbreak

The EVD outbreak affected the mental health of the Sierra Leone population not only through the impact of the disease itself, but also through perceptions of threat, loss of loved ones, loss of property and livelihood, rumours and uncertainty, loss of social support networks, inability to move freely, stigma and discrimination (Shultz *et al*., 2015; Jalloh *et al*., 2018; Weissbecker *et al*., 2018; Cénat *et al*., 2020).

A case study of 55 patients admitted to Ebola Treatment Units in Sierra Leone found that mental health-related symptoms were common, with low mood, appetite problems and anxiety most often reported (Weissbecker *et al*., 2018). In a national sample of Sierra Leoneans conducted just over 1 year into the EVD outbreak nearly 50% of respondents reported at least one symptom of anxiety or depression, with 16% meeting levels of probable PTSD diagnosis (Jalloh *et al*., 2018). High levels of distress were documented among Ebola survivors (Hugo *et al*., 2015; Ji *et al*., 2017; Jalloh *et al*., 2018).

The challenge in making use of these findings to inform mental health service development in Sierra Leone is that responses which are seen as pathological in a normal context may have been adaptive during the EVD outbreak (e.g. ‘excessive’ hand-washing; fear of contact with other people). Weissbecker *et al*. (2018) noted that the symptoms of anxiety and depression identified amongst patients admitted to Ebola Treatment Units were generally considered to be a normal response to a stressful and frightening situation, rather than signs of psychopathology. It is thus important to be cautious about drawing conclusions about mental illness within the Sierra Leone population from studies that were conducted in the midst of the EVD outbreak (Jalloh *et al*., 2018).

### Mental health issues in non-emergency contexts within Sierra Leone

Relatively few studies in Sierra Leone have investigated mental health disorders in the general population outside of an ongoing emergency situation. The small number of health-centre-based studies conducted have found that severe disorders such as psychosis and epilepsy were most common in those presenting at clinics, but interpretation of these studies is again confounded by the fact that they were completed immediately after the war (e.g. Jones *et al*., 2009) or during the EVD outbreak (Kamara *et al*., 2017).

To our knowledge, no systematic studies have been conducted on Sierra Leonean expressions of distress amongst adults. However, authors do refer anecdotally to common terms for distress and mental illness in Sierra Leone, particularly *crase* and *poil heart* (e.g. Jones, 2008; Harris, 2007; Harris *et al*., 2020). *Crase* is a Krio term indicating that someone is ‘crazy’, with *poil heart* (or *pwɛl at*) indicating ‘heavy heartedness’ (Cavallera *et al*., 2019: 121).

### Status of mental health provision

On the basis of population projections of need and mapping of current provision the mental health ‘treatment gap’ in Sierra Leone has been estimated to be 98% (Alemu *et al*., 2012). Formal mental health service provision is limited to one psychiatric hospital which receives referrals from provincial and district hospitals, NGO services and recent attempts to strengthen capacity at the primary care level through the training of mental health nurses (Harris *et al*., 2020). There is anecdotal evidence of widespread use of traditional healers and herbalists (Alemu *et al*., 2012), supported by a small number of studies (Jones *et al*., 2009), and a memorandum of understanding has been established between the Ministry of Health and Sanitation and the traditional healers' union in an attempt to coordinate care. Following the first national epidemiological mental health survey (Jensen and Nahim, 2002), a Mental Health Policy and National Mental Health Strategic Plan (2010–2015) was established (Alemu *et al*., 2012) and plans have continued to be developed but implementation has generally been weak (Harris *et al*., 2020).

Over 100 Community Health Officers have been trained in mhGAP, a package developed by the WHO to scale up prevention and management services for mental, neurological and substance use disorders in countries with low- and lower-middle incomes (WHO, 2016), but most are yet to receive any supervision or refresher training (Harris *et al*., 2020). A roll-out of Psychological First Aid training for primary healthcare workers (Sijbrandij *et al*., 2020) has lacked the follow-up that research has indicated is required to sustain a major uplift in capacity in this setting (Horn *et al*., 2019). In combination, a lack of sustained funding and a severe shortage of healthcare personnel renders universal mental health provision through a psychiatrically-trained cadre of health workers unfeasible to attain within the foreseeable future (Bah *et al*., 2018; McPake *et al*., 2019).

### Study aim

The study reported here was developed through discussions with stakeholders in Sierra Leone, including representatives from the Ministry of Health and Sanitation, non-governmental organisations and civil society bodies. It aimed to facilitate the understanding of the presentation of psychological distress in a manner that would inform the development of a culturally-valid and locally meaningful measure of mental health and psychosocial wellbeing and provide information to facilitate identification of resources, particularly community-based, relevant to prevention of, and response to, mental health issues in the Sierra Leone context.

This study did not investigate affected individuals' own reflections on their experiences of distress, nor explanatory models of distress (Okello and Ekblad, 2006; Okello and Neema, 2007; Abbo *et al*., 2008); the latter being the focus of a complementary study. Rather the focus was on signs of distress which are observable by those in contact with the individual, including family, friends and informal helpers. The term ‘sign of distress’ is used to indicate thoughts, feelings and behaviours perceived to indicate some form of emotional disturbance. The term ‘expression of distress’ is used to indicate a broader set of thoughts, feelings and behaviour which potentially occur together in an affected individual.

## Method

### Design

**Fig. 1.**
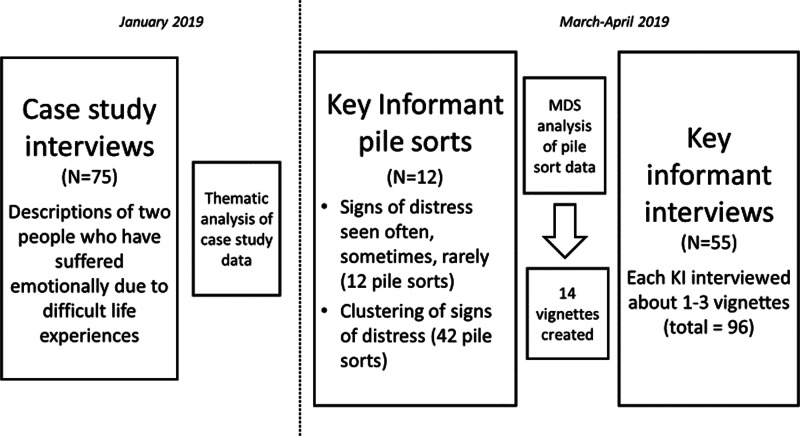
Design of study.

The study drew upon, and extended rapid ethnographic approaches suited to the investigation of local understandings of distress (e.g. Betancourt *et al*., 2009; Rasmussen *et al*., 2011; Keys *et al*., 2012; Bolton *et al*., 2013; Lee *et al*., 2018). The work included sequential case study interviews (Miller *et al*., 2006), pile sort exercises (analysed through use of multi-dimensional scaling, MDS) and in-depth interviews with key informants (KIs) as depicted in Fig. 1.

### Research team

The training and supervision of the research team was carried out by a Queen Margaret University researcher and coordination of logistical issues was conducted by a member of staff from the College of Medicine and Allied Health Sciences (COMAHS), University of Sierra Leone. The field researchers were all Sierra Leonean, aged between 20 and 30 years old, and were either recent university graduates or were in the final phase of their studies. They were from a number of ethnic groups, and spoke languages including Mende, Temne, Fullah and Limba as well as being fluent in Krio and English.

For case study interviews, a team of 11 field researchers (four female and seven male) participated in a 3-day training, and for the KI phase eight field researchers (four female and four male) participated in a 5-day training. The training consisted of sessions on research ethics and qualitative methods, plus intensive practical training in, and pilot testing of, the methods to be used. Eight of the field researchers had worked with the lead researcher (first author) previously on other research projects so were familiar with the types of methodologies used. Four of the field researchers (second, third, fourth and fifth authors) were involved throughout the project, including the data analysis.

### Case study interviews

Case study interviews involved asking the respondent to think of two people they know personally who have suffered emotionally because of difficult life experiences, one of whom has recovered and one has not. The respondent is then asked to tell the interviewer about the difficult life events each person experienced; how they were affected (thoughts, feelings and behaviour); signs that indicate that the first person has recovered; why they think the first person recovered, but the second is still having difficulties.

#### Selection of participants

Respondents were a convenience sample from five districts (Bo, Kailahun, Kambia, Kono and Western Area). The locations within each district were selected in collaboration with the Community Mobiliser at the District Health Management Team to reflect diversity in terms of factors such as religion, ethnicity, socio-economic status and main form of livelihood. Field researchers purposively sampled to ensure gender balance and a representative age range.

Case study interviews were conducted with 75 respondents, 15 in each of the five districts. In all districts except for Bo, eight men were interviewed and seven women; in Bo four men were interviewed and 11 women (total of 36 men and 39 women). Respondents ranged in age from 18 to 76 years, with a mean age of 42 years.

#### Process

Interviewers worked in teams of two (interviewer and note-taker/quality control) to carry out the data collection (Betancourt *et al*., 2009). Interviews were conducted in the respondent's preferred language, which was primarily (49) Krio. Non-Krio interviews were mainly conducted through a translator hired by the field researchers in the local area.

Interviews lasted around 40 min. Handwritten notes were taken during the interview, and reviewed afterwards by the interviewer and the note-taker to ensure that they accurately and comprehensively represented what was said by the respondent. The notes were typed up by the lead researcher at the end of each day.

#### Analysis of case study data

The case study data were entered into four Excel spreadsheets: events leading to distress; signs of distress; signs of recovery and factors perceived to contribute to or hinder recovery. This paper focuses only on the ‘signs of distress’ data, so only this aspect of the analysis is described.

The research team, consisting of the field researchers plus the lead researcher, combined those signs of distress which were the same. When items were worded differently but seemed similar, the group made decisions by consensus as to whether or not the items were the same or distinct from each other. If the group agreed that two items were the same, they selected the more appropriate wording to describe that sign of distress. This process resulted in 30 signs of distress being identified and described in both English and Krio.

### Pile sort exercises and interviews with key informants

Following the analysis of the case study data, pile sort exercises and in-depth interviews were conducted with KIs in four districts (Bo, Kambia, Kono and Western Area).

#### Selection of participants

During the first phase of the study, respondents had been asked to give names and contact details of people who they and others go to in their community when they are concerned about a person's emotional or psychological state (defined as problems of thoughts, feelings or behaviour). These individuals were approached in each district, and were asked to recommend others who were knowledgeable about the relevant topics (Betancourt *et al*., 2011) but did not deal with these problems professionally. Professionals were excluded because they tend to answer based on their training rather than reflect on local community and cultural perspectives (Lee *et al*., 2018).

Twelve KIs completed the pile sort exercise, and 55 participated in subsequent interviews. Respondent characteristics are described in Table 1.

**Table 1.** Characteristics of KIs

	*N*	Community leaders	Religious leaders	Traditional healers
Pile sort				
Bo	4	2 (both male)	1 (male)	1 (female)
Kambia	4	4 (all male)	0	0
Kono	4	3 (all male)	1 (male)	0
Western Area	8	0	0	8 (4 male, 4 female)
Interviews				
Bo	18	5 (3 male, 3 female)	3 (1 male, 2 female)	10 (6 male, 4 female)
Kambia	15	11 (6 male, 5 female)	3 (all male)	1 (female)
Kono	15	3 (2 male, 1 female)	1 (male)	11 (8 male, 3 female)
Western Area	7	4 (2 male, 2 female)	3 (1 male, 2 female)	0

#### Process

Interviewers worked in four teams of two. Each team conducted one pile sort exercise on the first day in each district, and two in-depth interviews on each subsequent day.

All pile sort exercises were conducted in Krio to ensure that the signs of distress were well understood by the KI. The subsequent interviews were conducted mainly in Krio (34), with most others conducted through a translator hired by the field researchers in the local area. The pile sort exercises took on average 1 h 17 min, and the in-depth interviews took on average 1 h 36 min.

#### Pile sort process and analysis

The 30 signs of distress identified from the case study interviews were written on cards in Krio and English. In the first district (Western Area) pile sorts were conducted as a group exercise with members of the traditional healers' association, but due to challenges with this approach those data have been excluded from the analysis reported here. In subsequent districts pile sorts were conducted with individual KIs, who were asked to sort the cards into three piles to represent those signs of distress they saw often within their community, those they saw sometimes and those they saw rarely. Twelve of this type of pile sort were conducted.

The interviewer then randomly chose a sign of distress from the ‘often’ pile and asked the KI to identify other signs which tended to accompany this (i.e. when they saw someone with this particular sign of distress, which other signs were often also present in the same person?). Once they had completed this, they were asked what this grouping of signs was called. The grouping task was repeated up to three times for each KI. A total of 42 of this type of pile sort was conducted. Each time, the KI could select from all the signs of distress, to allow some signs to appear in multiple categories (as recommended by Rasmussen *et al*., 2011). Pile sorting thus enabled preliminary identification of potential clustering of signs through participants physically grouping items and explaining relationships among them (Keys *et al*., 2012).

In each district, data from the clustering pile sort exercises were entered into an item-by-item matrix and combined to compute the percentage of pile sorts in which any two items had been placed on the same pile. This was used to produce a MDS two-dimensional map depicting the overall patterning of items in that district. Groupings of items suggestive of potential thematic clusters were identified and three vignettes produced in each district to reflect these (see Table 3 for examples of the vignettes).

#### In-depth interview process and analysis

The vignettes developed in each district were used as the basis for subsequent in-depth interviews with KIs. Each KI was interviewed about between one and three vignettes, depending on their availability and motivation. A total of 96 discussions of vignettes were held with the 55 KIs.

A vignette was read to the KI, who was asked whether they recognised this combination of signs. If so, they were asked what name this grouping of signs was commonly known by, and were then asked to describe: (a) the nature of the problem, including a description of symptoms; (b) the cause of the problem; (c) effects of the problem for the individual, family and community; (d) what people currently do about the problem and (e) what they think could or should be done about the problem (based on Lee *et al*., 2018). This paper focuses only on their recognition and naming of these groupings of signs.

Handwritten notes were taken during each KI interview, following which the interviewer and note taker worked together to create a ‘condensed verbatim transcript’ (following Columbia Group for Children in Adversity, 2011; Child Resilience Alliance, 2018). Each transcript was reviewed and typed by the lead researcher, and written and verbal feedback given to the field researchers on an ongoing basis.

The lead researcher developed a coding scheme based on the five domains around which the interview questions were organised. She coded the data into these categories using the on-line analysis package, Dedoose. The data related to signs of distress were reviewed in relation to the 30 signs previously identified to add more detail to descriptions of each sign, and identify potential additional signs. The data relating to the grouping of signs, and the names given to different types of expressions of distress were analysed thematically.

### Ethical considerations

Respondents were read an information sheet about the study and gave verbal consent to be interviewed, which was documented by the field researchers. For the case study interviews, names were not recorded. The names and contact details of KIs were recorded in case follow-up interviews were necessary; these were kept separately from the transcript of each KI's interview.

For both types of interviews, we did not seek information about the specific problems of individual respondents and interviewers were trained to redirect any discussion about personal experience back to the community in general. Interviewers were also trained in how to respond if a respondent became upset; however, during data collection this did not prove to be necessary.

This study was approved by the Queen Margaret University Edinburgh Research Ethics Committee and by the Office of the Sierra Leone Ethics and Scientific Review Committee, Ministry of Health and Sanitation. All field researchers were trained in ethical issues relating to the research, and signed a Code of Conduct.

## Results

### Signs of distress: frequency and commonality

The signs of distress identified from the case study interviews are summarised in Table 2. The third column summarises the data from the case study interviews: the number of times each sign of distress was mentioned is listed and the percentage of cases in which each sign was noted. The fourth column gives the mean score for how often KIs reported encountering each sign of distress, with scoring given as 3 for very often, 2 for sometimes and 1 rarely. Items are ordered by descending pile sort mean.

**Table 2.** Summary of signs of distress

Krio	English	*N* (%)	Pile sort mean
*Nɔ gladi*	Feeling or looked sad and unhappy; discouraged; felt disappointed	69 (*46.0*)	2.83
*Fɔstret*	Frustrated	33 (*22.0*)	2.75
*Nɔ de gri fɔ it*	Loss of appetite/stops eating	26 (*17.3*)	2.75
*De mɛmba bɔku*	Thinks too much or overthinking	4 (*2.7*)	2.75
*Gɛt wam at; vɛks; kɔs; nɔ gɛt rɛspɛkt fɔ pipul dɛm*	Hot tempered or angry; arrogant; rude or abusive to people^a^	42 (*28.0*)	2.73
*Kip to iŋsɛf*	Stays indoors or isolates self; disconnects from people, stops going to public gatherings including mosque or church	61 (*40.7*)	2.67
*Dɔti*	Appears dirty or untidy (including urinating or defaecating on self); may eat dirty food from street or dustbin; picks up dirty things	57 (*38.0*)	2.58
*Smok jamba; tek drɔgs; drink rɔm tu mɔs*	Smokes marijuana; takes drugs; drinks alcohol	37 (*24.7*)	2.58
*I kɔnfyuz ɛn nɔ de pe atɛnshɔn*	Loss of concentration; felt or seemed confused; sometimes unable to recognise or remember people they knew well.	32 (*21.3*)	2.58
*Di pɔsin de fil se dɛm dɔn bitre am ɔ pipul dɛm het am, di pɔsin de blem ɔda pipul dɛm*	Feels betrayed; feels whole world is against her or him; feels hated by all in community; blames others for what happened.	27 (*18.0*)	2.58
*De tink se dɛm swɛ am ɔ dɛm wich am*	Thinks is bewitched/cursed	17 (*11.3*)	2.58
*Fil lɛk I nɔ gɛt yus; fil lɛk I dɔn fel na layf*	Believes they are useless to self and others; feels worthless and/or like a failure	15 (*10.0*)	2.50
*De gladi fɔ smɔl tɛm ɛn bifo u de no, I dɔn vɛks bak*	Moody/mood swings	3 (*2.0*)	2.50
*Di pɔsin de fil se dɛm nɔ wan no but am*	Feels lonely; feels neglected/rejected/unloved/abandoned	30 (*20.0*)	2.42
*De tɔk bɔku ɛn pipul dɛm nɔ de ɔndastan*	Talks a lot and/or speech makes no sense	16 (*10.7*)	2.42
*Nɔ de ebul slip*	Cannot sleep and/or has nightmares	12 (*8.0*)	2.42
*Tɔk to iŋsɛf ɛn ɔda tin dɛm*	Talking to self and/or inanimate objects (e.g. trees); sings, shouts or laughs to self	60 (*40.0*)	2.33
*Nɔ gɛt op*	Loss of hope; feels hopeless	45 (*30.0*)	2.33
*De cray ɔl tɛm*	Often crying	33 (*22.0*)	2.33
*Kwayɛt ɛn I nɔ de gri tɔk*	Stays quiet, unwilling to talk or respond to attempts to talk to them	26 (*17.3*)	2.33
*Tɔmɛnt ɛn de waka bɔku*	Moving from one place to another; restless, cannot stay still	21 (*14.0*)	2.33
*De fɛt ɔ I agrɛsiv to ɔda pipul dɛm; de tink fɔ du bad ɔ kil ɔda pipul dɛm*	Violent or aggressive to people (stoning, fighting, beating wife, taking people's food); has thoughts of harming or killing others (e.g. wife); destroys things (or wants to)^b^	48 (*32.0*)	2.20
*Lɛf fɔ go wok ɛn lɛf fɔ go skul*	Quits whatever constructive activity he/she was involved in (work, school, farming, business)	20 (*13.3*)	2.17
*De fil shem ɔ gilti*	Feels ashamed/guilty	15 (*10.0*)	2.17
*Slip na trit*	Sleeping in public places such as in the street.	8 (*5.3*)	1.92
*I de fred pa iŋsɛf ɛn wɔri*	Feels afraid or worried	9 (*6.0*)	1.83
*Nɔ de tek tritmɛnt*	Refuses to engage with treatment and/or refuses to take medication^a^	3 (*2.0*)	1.82
*I de si ɛn yɛri tin dɛm we pipul dɛm nɔ de si ɔ yɛri*	Sees people others don't see and/or hears voices others don't hear	2 (*1.3*)	1.75
*Wan du iŋsɛf bad ɔ kill iŋsɛf*	Thoughts of/attempts suicide (e.g. drinking poison); physically harms self (e.g. pinches skin, pulls hair)	55 (*36.7*)	1.50
*De ɔndrɛs na pɔblik*	Removes clothes in public	8 (*5.3*)	1.50

a Missing data = 1 for pile sort data.

b Missing data = 2 for pile sort data.

### Signs of distress: item grouping and validation of clusters

During the pile sort exercise, 27 of the 42 groupings of signs produced were judged by the KI to have no local name. Of those which were given a name, three were named *frustrate*, three were named *crase*, with others assigned a diverse range of terms.

On presentation of the vignettes to KIs, on only three of 96 occasions did KIs report that they did not recognise that grouping of signs. Of the other 93, in 13 cases the KI said there was no name for that group of signs, and in another 10 cases they said there was no standard or agreed name but they referred to it with some general term indicating that the person had a problem, such as unhappy, stressed, ‘abnormal or semi-normal’ or not well. Five KIs gave the grouping of signs a name which related to some supernatural cause; three KIs gave names that were a judgement on the person's behaviour (e.g. wicked), and two gave names relating to the circumstances leading to the distress (e.g. failure).

The majority of names were given related to some kind of emotional or psychological state. The most common description (in 20 cases) was *craseman* (Krio) or another language descriptor with the same meaning. Also common (10) was frustrated or *fɔstret* in Krio (although *fɔstret* was often combined with another name, as described below). Less common but also used were *aflahun* (3); traumatised (4); stress (5); not normal (3); *de posɛn wan go off* (Krio) (2).

Sometimes (8) people would say ‘X or X’, indicating that the two names for the problem were interchangeable. This applied to frustrated/traumatised; frustrated/madness; *aflahun*/*craseman*; *aflahun*/*mumu*; mentally ill/*fool*; *pwɛl heart*/*frustrate*; discouragement/frustration; stress/traumatised.

Analysis of the names given to each of the groupings similarly shows a lack of agreement within the KIs inconsistent with these being considered valid and reliable clusters. In several cases the KI said no name was given, or that there was no agreed name for this type of problem. Some examples are given below (Table 3).

**Table 3.** Examples of divergent naming of groupings of signs of distress across participants

Vignette	Names given
Imagine you meet someone who has recently changed their behaviour, and sits alone for a lot of the time, just thinking. Their mood is unstable – sometimes they're OK then sometimes they are silent. They say they feel hopeless about the future. Friends and family have noticed that the person has stopped taking care of themselves – they are dirty, and their clothes and hair are untidy.	Mad or *crase* (4); frustrated/traumatised; no name; frustrated; *aflahun* (3); *fool*; poverty
Imagine you meet someone who has recently changed their behaviour. They seem to be thinking too much, and people have noticed that they are drinking and smoking more than they used to. They are not able to concentrate on what people say to them because their mind is always on their own issues, and they forget things they have been asked to do. They still take care of themselves – they are clean and tidy, and they are never violent or abusive to people, they just seem to be lost in their own thoughts for most of the time.	No name (2); *de posɛn wan go off*; the head box up; traumatised; *mamuu* (mental person); *crase* (2); stress; thinking sickness; frustration (2)
Imagine you meet someone who has recently begun talking to themselves and to objects such as trees. They appear to see people that others cannot see, and to hear things that others cannot hear. They are very sad, and they cry a lot of the time. They have almost stopped eating and say they feel useless to society and hopeless about the future. They do not drink alcohol or take any kind of drugs, so their family cannot understand why they have started behaving this way.	*Crase* (5); devil sickness (3); traumatised; not normal; no name (2)

As a final step of triangulation, MDS analysis of the aggregate sorting of the 30 signs across all four districts confirmed no consistent clustering of items, with a goodness of fit score of >0.15 indicating unclear multi-dimensionality (see online Supplementary figure). No major trends were identified in terms of location (District) or gender of KI.

## Discussion

This study identified broad consensus around common signs of psychological distress in Sierra Leone but a lack of consistency in how these signs are understood to cluster in people's experience. When respondents grouped signs together there was often no name – or no agreed name – for the grouping they described. *Crase* was the only term used with any frequency but, although this was generally linked to active, psychosis-related signs of distress, reference was also frequently made to a range of other thoughts, feelings and behaviours.

The lack of recognised and named syndromes of distress in Sierra Leone is unexpected, given the findings of studies in other cultural settings which used similar methodologies (e.g. Rasmussen *et al*., 2011; Ventevogel *et al*., 2013). However, when the findings were discussed with Sierra Leonean members of the research team and national experts (e.g. members of the Mental Health Coalition-Sierra Leone) they did not express surprise. Their understanding was that in Sierra Leone, people are seen as either *crase* or well, with some recognition also of a temporary ‘not normal’ state in response to difficult events. They concurred with participants in the study that various words could be used to describe moderate distress but there were many different possibilities with no agreed definitions. Diversity within and between languages in Sierra Leone may contribute to the lack of agreed names for different forms of psychological distress. The most common language used throughout the country is Krio, but most people also speak one of the 23 local languages, and outside Freetown the local language is often dominant. Even within local languages there are different dialects, each of which may have a different term for a specific expression of distress.

However, linguistic issues in themselves do not account for our findings; there is also a conceptual issue relating to a generalised sense of mental ill-health which includes a wide range of interrelated expressions and idioms. This is not a unique finding. In Uganda, for example, traditional healers were found to have little interest in naming the illnesses they are asked to treat, and ‘would sometimes lump all mental illnesses together because they found it difficult to name them individually’ (Abbo *et al*., 2008: 139). These views of mental illness have more in common with a continuum of distress model than one focused on discrete categories.

The recent *Lancet Commission on Global Mental Health and Sustainable Development* (Patel *et al*., 2018) emphasised such a dimensional approach to mental health, which ‘lends itself to identifying public policies that promote and protect mental health for all people, irrespective of the presence of a mental disorder, much more than the restrictive concept of dividing people into those who do not have a mental disorder and those who do’ (p. 33). While the authors recognise that psychiatric classification systems have clinical utility, a categorical approach to mental health, whether this is based on psychiatric classifications or local categories of distress, risks the reification and medicalisation of these categories (Ventevogel, 2016; Miller, 2017). An example of this is the transformation by humanitarian organisations in Liberia of a broad, diverse concept of physical and emotional suffering known as ‘*Open Mole*’ into a local idiom of PTSD-related mental illness (Abramowitz, 2010).

An alternative approach is to see any discrete disorders that can be identified in a community as ‘heuristic concepts used pragmatically to bring order to chaotic and disturbing experiences and to assist in the quests for meaning and solutions to end suffering’ (Ventevogel, 2016: 90). Kirmayer *et al*. (2017) encourage us to ‘[t]ake a step back from medicalising distress in terms of discrete disorders and look at it as a sign that something is wrong in the person's lifeworld’ (p. 167). Such a view of mental (ill)health can empower a wide range of service-providers to offer support to those experiencing psychological distress; better meet the needs of those with mental health problems; and ensure better use of resources to promote mental health and address the needs of those experiencing psychological distress (Patel *et al*., 2018).

Primary healthcare workers in global settings often do not use the mental health categories in which they have been trained because they do not fit with their experience and are not useful in understanding and responding to the forms of distress with which people present (Heath, 1999; Thangadurai and Jacob, 2014). Jacob and Patel (2014) suggest that rather than responding to this with further training, management guidelines or diagnostic algorithms – which currently characterises approaches to strengthening mental health provision in Sierra Leone – it may be more helpful to consider whether the mental health paradigm on which such training is based is appropriate for the setting. Faregh *et al*.'s (2019) study of cultural and contextual challenges in mhGAP training and implementation identified cultural differences in explanations of and attitudes towards mental disorder as one of the six main challenges, and recommended that mhGAP training addresses the specific kinds of problems, modes of clinical presentations and social predicaments seen in the local population. Our findings can contribute to the process of adapting mhGAP materials to include the signs of distress that people are likely to present with in the Sierra Leone context.

Furthermore, the emphasis on diagnostic categories has hampered preventative psychiatry and the capacity for early diagnosis (McGorry and van Os, 2013). As noted earlier, while there has been some progress in strengthening supports for those most severely affected by mental health problems in Sierra Leone, little attention has been paid to those who experience mild-to-moderate distress (Harris *et al*., 2020). This is despite the fact that common signs of distress such as anxiety or low mood are associated with more total disability at a population level than diagnostically defined mental disorders (Das-Munshi *et al*., 2008) and a proportion of those experiencing mild-moderate distress will go on to develop more severe mental health problems if they do not receive the necessary support. Early identification of onset of problems leads to better outcomes, which is especially important in a setting such as Sierra Leone, where there are such limited formal mental health services. The findings of this study can inform training of non-specialists in how to recognise mild-moderate forms of mental health problems as they are presented in their context.

Recent analysis has highlighted the implausibility of addressing mental health needs through the strengthening of the healthcare workforce in Sierra Leone (McPake *et al*., 2019). Our findings point to the need to strengthen the ability of informal helpers at the community level to provide effective social and emotional support, as well as referring people for different types of help. Given the constraints on the formal health system, this must be a central feature of any feasible approach to prevention of, and response to, mental health issues in Sierra Leone.

**Fig. 2.**
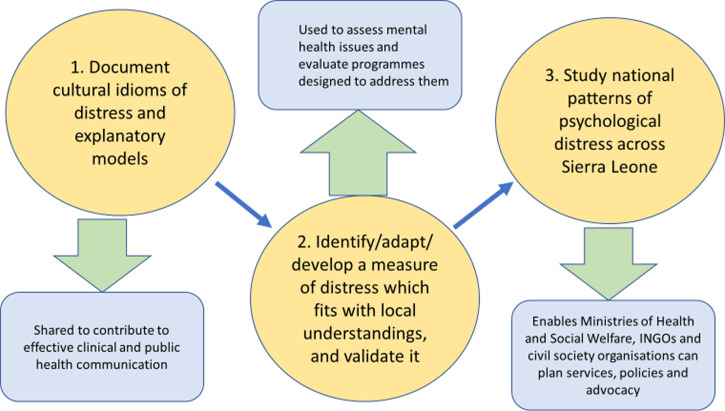
Key steps in building on understanding cultural expressions of distress in Sierra Leone.

The next steps of our work (see Fig. 2) – supported in agenda setting consultations with the Ministry of Health, National Statistical Office, the Mental Health Coalition-Sierra Leone and other stakeholders – are to develop a tool to measure psychological distress as it is expressed in the Sierra Leone context. Once the reliability and validity of the scale are established it will be used to conduct a national survey of psychological distress. Findings will be shared with government bodies and others, to identify ways for these data to inform development of formal and informal supports for those affected by mild, moderate and severe mental health problems: plotting a feasible, empirically-based and culturally-grounded strategy for mental healthcare in Sierra Leone.

## Limitations

The two main challenges experienced were the identification of suitable KIs, and the process of conducting the pile sort exercises. It quickly emerged that community respondents had been reluctant to share names and contact details about informal healers, so we changed the strategy of identifying KIs to asking community leaders for recommendations of who we should speak to, and then using the snowball methodology more often. In terms of the pile sort methodology, initial plans to conduct this in a group format (as in Rasmussen *et al*., 2011) were adjusted after the process was dominated by one or two people in a piloting of the approach with a group of traditional healers in Freetown. Pile sorts with individual KIs proved much more effective.

Additionally, with some statistical conditions for MDS being only partly met (e.g. independence of scoring), analysis of potential clustering of items by this method needs to be considered exploratory in nature, and a source of triangulation with thematic analysis rather than robust in its own right.

It should also be noted that the relative youth and highly educated status of the field researchers will have had some influence on their interactions with both community members who participated in case study interviews, who were of a range of ages and backgrounds, and KIs, who tended to be older than the field researchers. It was important, especially for the KIs, that the field researchers were Sierra Leonean. One traditional healer stated that he would not have been willing to be interviewed by someone from outside the country because he felt his experience was unlikely to be respected by someone who did not understand the context.

## Conclusions

Studies of mental health in Sierra Leone have been dominated by investigations during or in the immediate aftermath of crisis, often using the framework of psychiatric categorisation. This study engaged community members and non-clinical KIs who were recognised as a source of emotional and psychological support. There was broad consensus around common signs of psychological distress but a lack of consistency in how these signs are understood to cluster. Although *crase* is used as term to describe mental ill-health with some frequency, it is used inconsistently, in association with a range of other signs, and in describing a minority of presentations of distress. The symptom-based – rather than syndrome-based – approach to the assessment and treatment of distress encouraged by these findings is conceptually-aligned with calls for a dimensional approach to address global mental health (Patel *et al*., 2018). Moreover, this approach is both culturally-grounded and – in a context where healthcare human resources are insufficient for reliable provision through formal health providers – suited to the engagement of community-based supports.

## Supplementary material

For supplementary material accompanying this paper visit http://dx.doi.org/10.1017/gmh.2020.12. click here to view supplementary material
