# Supplementary material for: Expressions of psychological distress in Sierra Leone: implications for community-based prevention and response
Source: Glob Ment Health (Camb). 2020 Jul 29;7:e19. doi: 10.1017/gmh.2020.12 (PMC7443608; doi:10.1017/gmh.2020.12)

Supplementary Figure. Two Dimensional MDS Plot of Relationships Between Numbered Items Indicating Lack of Consistent Clustering


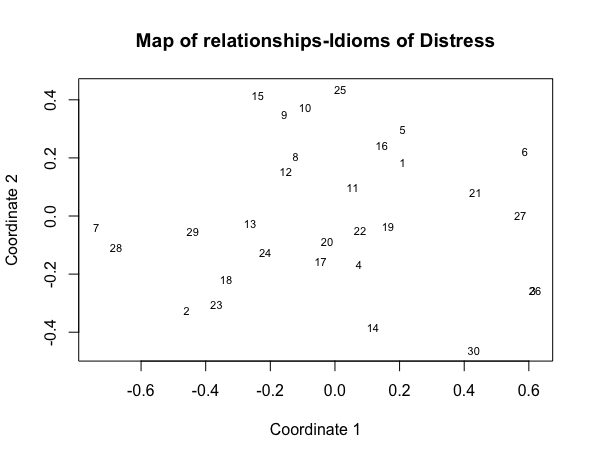

Supplement: Supplementary file 1 [file S2054425120000126sup001.docx]
